# Utility and safety of a novel surgical microscope laser light source

**DOI:** 10.1371/journal.pone.0192112

**Published:** 2018-02-01

**Authors:** Taku Sato, Mudathir S. Bakhit, Kyouichi Suzuki, Jun Sakuma, Masazumi Fujii, Yuta Murakami, Yuhei Ito, Tetsuo Sugano, Kiyoshi Saito

**Affiliations:** 1 Department of Neurosurgery, Fukushima Medical University, Fukushima, Japan; 2 Department of Neurosurgery, Fukushima Red Cross Hospital, Fukushima, Japan; 3 Kyoto Office, Mitsubishi Electric Engineering Co., Ltd, Kyoto, Japan; Waseda University, JAPAN

## Abstract

**Objective:**

Tissue injuries caused by the thermal effects of xenon light microscopes have previously been reported. Due to this, the development of a safe microscope light source became a necessity. A newly developed laser light source is evaluated regarding its effectiveness and safety as an alternative to conventional xenon light source.

**Methods:**

We developed and tested a new laser light source for surgical microscopes. Four experiments were conducted to compare xenon and laser lights: 1) visual luminance comparison, 2) luminous and light chromaticity measurements, 3) examination and analysis of visual fatigue, and 4) comparison of focal temperature elevation due to light source illumination using porcine muscle samples.

**Results:**

Results revealed that the laser light could be used at a lower illumination value than the xenon light (p < 0.01). There was no significant difference in visual fatigue status between the laser light and the xenon light. The laser light was superior to the xenon light regarding luminous intensity and color chromaticity. The focal temperature elevation of the muscle samples was significantly higher when irradiated with xenon light in vitro than with laser light (p < 0.01).

**Conclusion:**

The newly developed laser light source is more efficient and safer than a conventional xenon light source. It lacks harmful ultraviolet waves, has a longer lifespan, a lower focal temperature than that of other light sources, a wide range of brightness and color production, and improved safety for the user’s vision. Further clinical trials are necessary to validate the impact of this new light source on the patient’s outcome and prognosis.

## Introduction

Continued technological advances regarding the optics, magnification, and illumination of surgical microscopes have resulted in operation at increased levels of magnification, requiring powerful light sources that generate heat. As the distance from the light source to the tissue decreases, the magnitude of the radiant heat on the tissue increases [1–3]. Tissue injuries caused by the thermal effects of xenon light microscopes have previously been reported [4–8]. Thus, the development of a safe light source is a necessity. Furthermore, the emission spectrum of xenon lamps has strong energy intensity in the ultraviolet wavelengths [1]. Exposure to the ultraviolet radiation induces functional changes in keratinocytes and immune cells that lead to skin cancer, supported by strong epidemiological and molecular evidence demonstrating a strong causal link between the ultraviolet exposure and all forms of skin cancer [9]. Considering the points mentioned above, we developed a new laser light source that can be integrated with conventional surgical microscopes [10].

## Materials and methods

**Fig 1:**
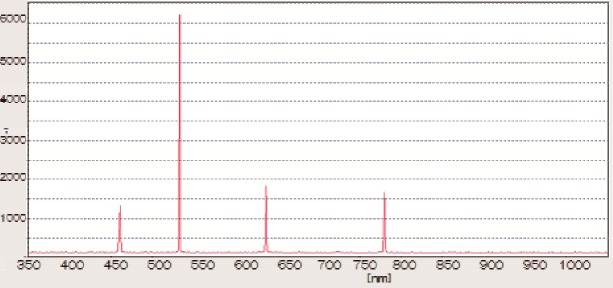
Laser light spectrum. Four wavelengths: 464 nm (blue), 532 nm (green), 640 nm (red), and 785 nm (near-infrared) were used. There were no ultraviolet wavelengths.

**Fig 2:**
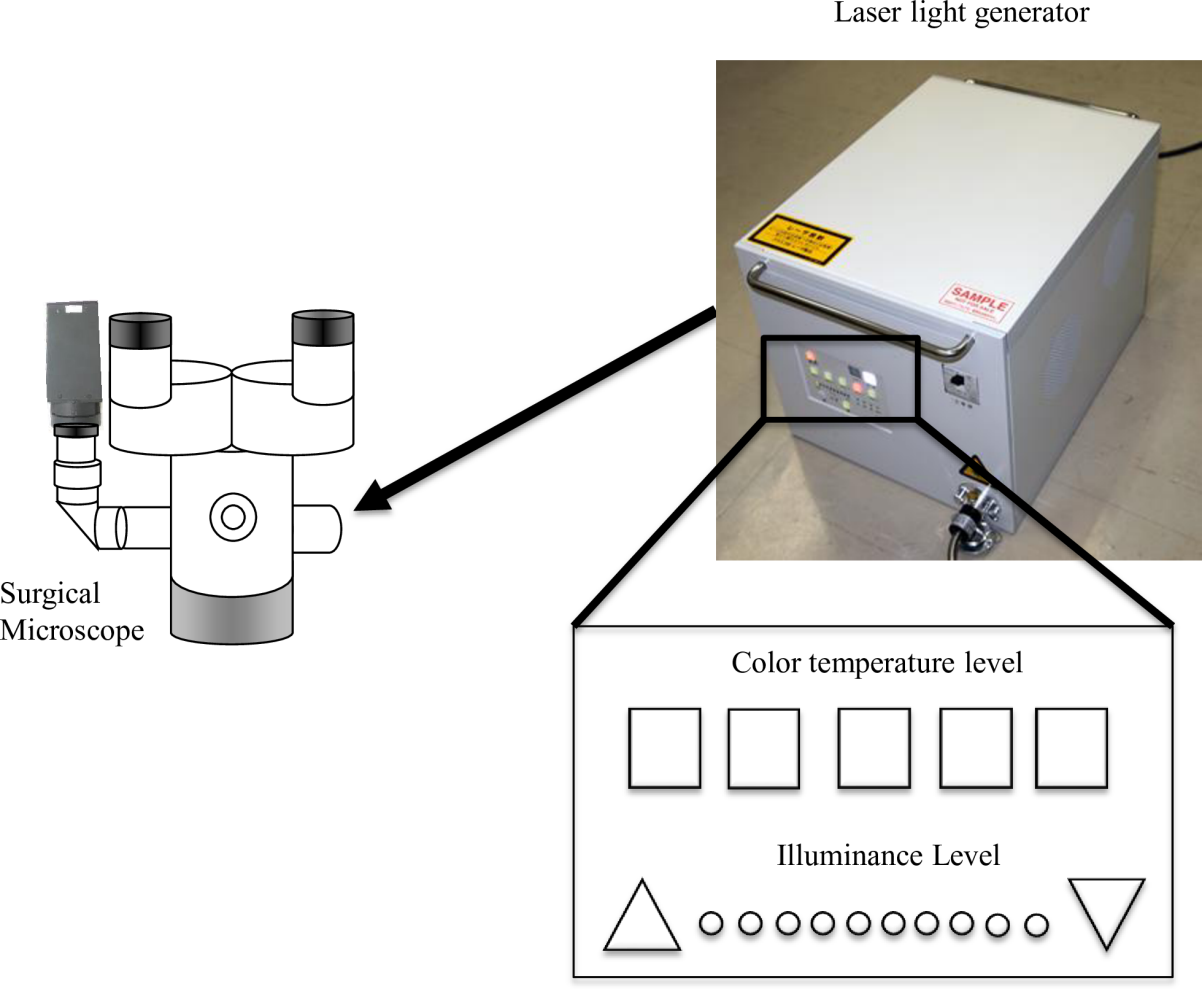
Laser microscope setup details. The diagram shows a conventional surgical microscope integrated with a laser light source from a laser generator allowing adjustment of color temperature levels and illuminance levels.

We developed a new laser light source for surgical microscopes. This laser illumination system does not consist of a complex line spectrum, as do other light sources. The illumination wavelength includes four bands, all of which combine to produce a white color: 464 nm (blue), 532 nm (green), 640 nm (red), and 785 nm (near-infrared) (Fig 1). This spectrum was optimally narrowed to avoid the harmful high-energy ultraviolet spectrum. The near-infrared 785 nm wavelength was included for indocyanine green excitation during fluorescence image-guided surgery. The new setup allowed the user to adjust two illumination variables: 1) the color temperature, with five different levels in Kelvin (K) units, and 2) the illuminance (light falling on the surface), with 10 different levels of lux (lx) units (Fig 2) [5]. For experimental purposes, the laser light’s color temperature was preset to 7000 K, which is closest to daylight color temperature (6500 K or D65) [11]. The upper five of ten levels of illuminance were not included in this study because their values were too high illuminance to use in the usual surgery. The spatial coherence of the four laser beams was reduced using a diffuser and an integrator rod. Similar methods to minimize laser beam coherence have been previously reported [12,13]. The aim of light coherence reduction is to avoid illumination of the harmful coherent laser light on the surgeon’s or the assistant’s eyes and the patient’s brain tissue [14]. The safety of laser illumination was assured by terminating the light source emission automatically when the connection cable was not attached to the laser light source. We don’t use shielding equipment for the eye protection from the laser light. Another institute had evaluated the eye protection matter from the laser light source. They performed a simulation of the photochemical and heat influence based on a Monte Carlo modeling. Even if a light diffusion reflection from the brain surface was observed under the maximum power light for a long time, the maximum exposure tolerance wasn’t exceeded. Also, even if a reflexed light from a surgical instrument was observed directly, the rapid close eye or the avoidance of the long time observation had ensured enough safety (Hazama H et al: [The development of a novel medical laser source using the three primary color lights and near-infrared laser light.], presented at the 35th Conference of Journal of Japan Society for Laser Surgery and Medicine, Tokyo, Japan, 2014). Accordingly, the laser light source was approved for employment as a medical instrument in Japan.

In the current study, three different commercially available surgical microscopes were used: 1) Leica OH-6 (Leica Microsystems GmbH, Wetzlar, Germany), 2) Mitaka MM80/YOH (Mitaka Kohki Co., Ltd., Mitaka, Japan), and 3) Zeiss OPMI^®^ PENTERO^®^ 900 (Carl Zeiss, Jena, Germany). Since the Zeiss microscope did not support the installation of the new laser light source, only two microscopes were tested with the laser light (Leica and Mitaka), and all three were tested with their existing xenon lights. The experimental protocol was reviewed and approved by a local independent ethics committee (No. 2470), and all volunteers (aged 20–39 years) gave their written informed consent after being provided with a detailed explanation of the study. There was no history of ocular disease or surgeries in any of the volunteers. The volunteers for the luminance test were doctors, nurses, or medical students. Only doctors participated in the visual fatigue status assessment.

The temperature and humidity of the examination room were maintained at 23°C–25°C and 43%–45%, respectively. Throughout the experiment, the laser or xenon light emitted from the microscope had a focal length of 285 mm. The study methods are detailed below.

### Luminance visual comparison test

Luminance refers to the brightness of the surface in candela per square meter (cd/m^2^) [15]. Fourteen volunteers each used a xenon light surgical microscope with an illuminance of 180,000 lx for 3 min. Then, the same volunteer used a laser light surgical microscope for 3 min, and the chosen five of ten levels of illuminance were randomly changed and not revealed to the volunteer (Fig 2). The volunteers were asked which level of laser light was similar to the previous xenon light experience. The illuminance levels were confirmed using a TL-1 illuminance meter (Konica Minolta Inc., Tokyo, Japan). A control illuminance of 180,000 lx was chosen for the xenon microscope because we considered this satisfactory for illuminating deep surgical locations.

### Luminance and chromaticity measurements

The brightness of both light sources was assessed by measuring the luminance values. The color quality of the illuminated surfaces was checked by applying the International Commission on Illumination CIE1931 color space chromaticity diagram and comparing the xenon and laser light color ranges [11,16]. The luminance and chromaticity (x and y coordinates) were measured using an LS-110 luminance meter (Konica Minolta Inc., Tokyo, Japan), and a test color sample chart was used. The luminance values and chromaticity (x and y coordinates) for each illuminated color test sample were recorded, and a brightness radar chart and a chromaticity color range diagram were constructed accordingly.

### Visual fatigue status assessment

The effect of the visual display of surgical microscopes on the user’s eyes has been reported in the literature using various indicators [17,18]. Although some of these methods are subjective, they remain in use to assess eye fatigue. The methods used to assess eye fatigue in the current study consisted of three parts: 1) assessment of eye accommodative function, 2) evaluation of lacrimal secretion, and 3) completion of a questionnaire regarding 15 visual fatigue-related symptoms. We analyzed the differences in eye status before and after microscope usage. Ten healthy volunteers had performed a microvascular anastomosis simulation model with silicone tube using either a laser or a xenon microscope light source randomly at 120,000 lx for one hour for two consecutive days. The type of light source was not revealed to the volunteers.

#### Accommodative function of the ciliary muscles

Measurement of the accommodative function of the ciliary muscles has been proposed by ophthalmologists as an objective assessment of visual fatigue [18–20]. The spectral power of accommodative microfluctuation was analyzed by fast Fourier transformation, dividing it into a 1.0–2.3 Hz high-frequency component (HFC) and a low-frequency component of less than 0.6 Hz [21]. The spectral power values were then converted to common logarithmic values. The sum of the logarithmic values between 1.0 and 2.25 Hz was defined as the spectral power value of the HFC [19]. The method of Kajita et al. was used in the current study [19]. They proved that the HFC in the low accommodation group (HFC-1) of accommodative microfluctuation could be used as a tool for diagnosing visual fatigue. Furthermore, Kajita et al. found that the spectral power value of HFC-1 was higher in subjects with asthenopia than in healthy subjects, with values of 60–70. Both eyes of each volunteer were tested. An accommodative microfluctuation analysis system (Auto Refractometer ARK-1s, NIDEK Co. Ltd., Gamagori, Japan) and software (AA-2, NIDEK) were used to calculate the spectral power value of HFC-1 of the microscope user’s eye. The volunteers’ HFC-1 readings before and after using the microscope were calculated as mean ± SD.

#### Strip meniscometry

Estimation of tear volume is regarded as essential for the diagnosis of dry eye syndrome [22]. Visual fatigue was evaluated using a strip meniscometry test. Sterilized strips of filter paper (SMTube, Echo Electricity Co., Ltd., Fukushima, Japan) were placed in the lateral canthus away from the cornea and kept in place for five seconds. Readings were reported as mean ± SD millimeters of wetting for 5-second values [22,23].

#### Questionnaire

The changes in symptoms of ocular surface dryness, fatigue, and discomfort were evaluated using visual analog scale (VAS) scores [17]. Changes in each of the following symptoms were evaluated: a feeling of heat, itchiness, foreign body sensation, tearing, frequent blinking, congestion, eye pain, droopy eyelid, dry eye, blurred vision, diplopia, difficulty in sustaining visual activity, seeing flashes, obscured view, and headache. The VAS score was calculated from a point marked on a 10-cm-long line representing the severity of the symptoms (minimum, 0 cm; maximum, 10 cm) (S1 File). Volunteers placed a mark corresponding to the severity of each symptom before and after using the microscopes.

### Measurement of temperature change

Six pairs of porcine muscles (2 cm × 3 cm × 3 cm) were prepared, and one of each pair was used as a control. The other muscle piece was illuminated with laser light or xenon light at 150,000 lx for 30 min. Before switching between laser light and xenon light, the temperature drop was confirmed for each muscle. The temperature of each pair of muscles was measured every 2 min by a thermographic camera (CPA-0304, CHINO Corporation, Tokyo, Japan). The temperature of the illuminated muscle was subtracted from the temperature of the control sample, and the change in temperature was documented.

### Statistical analysis

Data are presented as mean ± SD. The Wilcoxon signed rank test was used to compare pairs of dependent samples. Results were considered significant if p < 0.05.

## Results

### Luminance visual comparison test

Five volunteers stated that the laser light level of 180,000 lx was equivalent to 180,000 lx xenon light. Six reported that the laser light equivalent was 120,000 lx, two stated 148,000 lx, and one stated 220,000 lx (S2 File). These results suggest that laser light at lower illuminance values produces the same luminance effect as xenon light at higher illuminance values (p < 0.01).

### Luminance and chromaticity measurements

**Fig 3:**
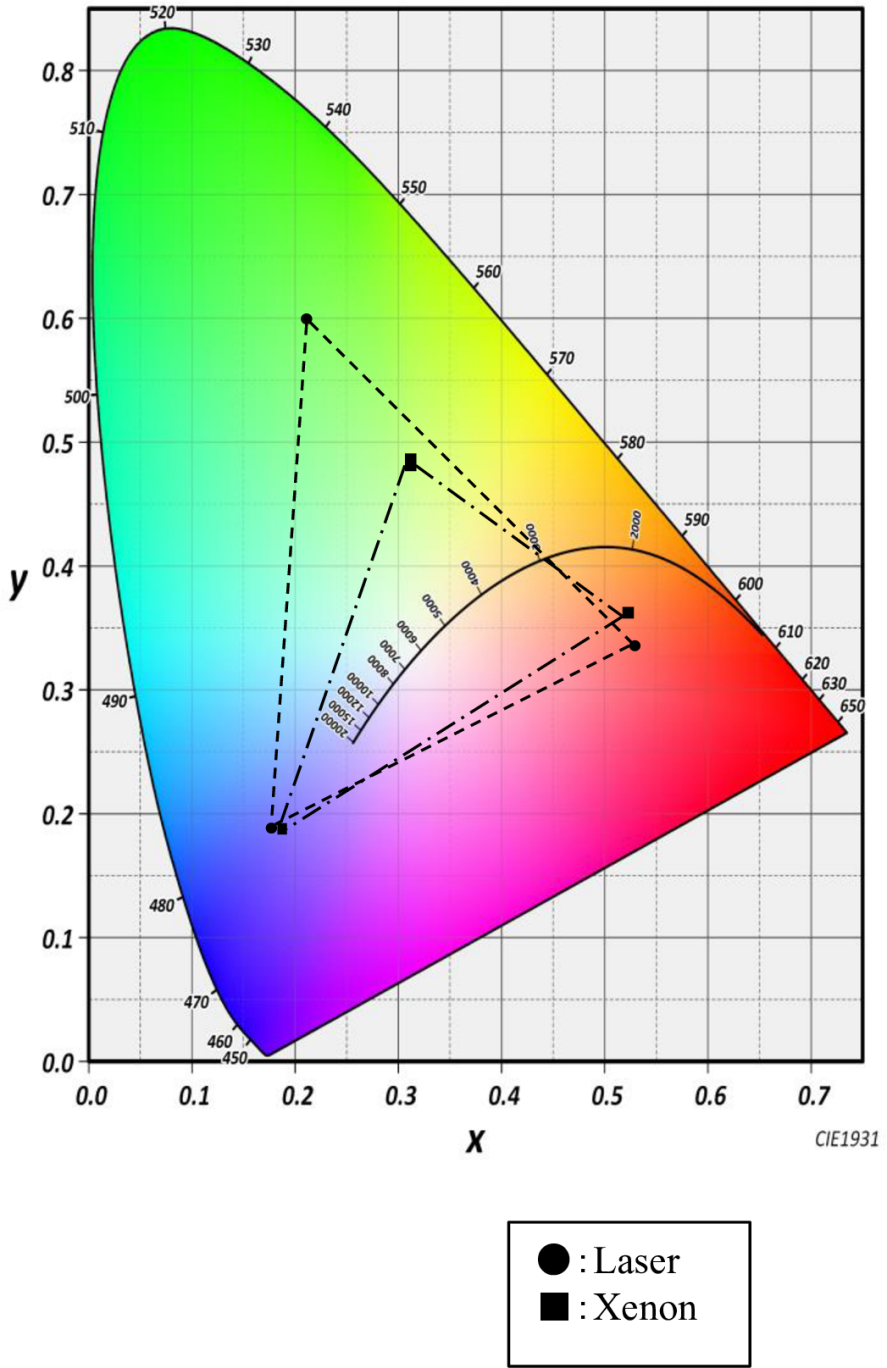
CIE1931 chromaticity diagram showing the range of colors of both xenon and laser light. The color coordinates were measured by a luminance meter. The laser light shows a wider range than xenon light in the red and green zones.

**Fig 4:**
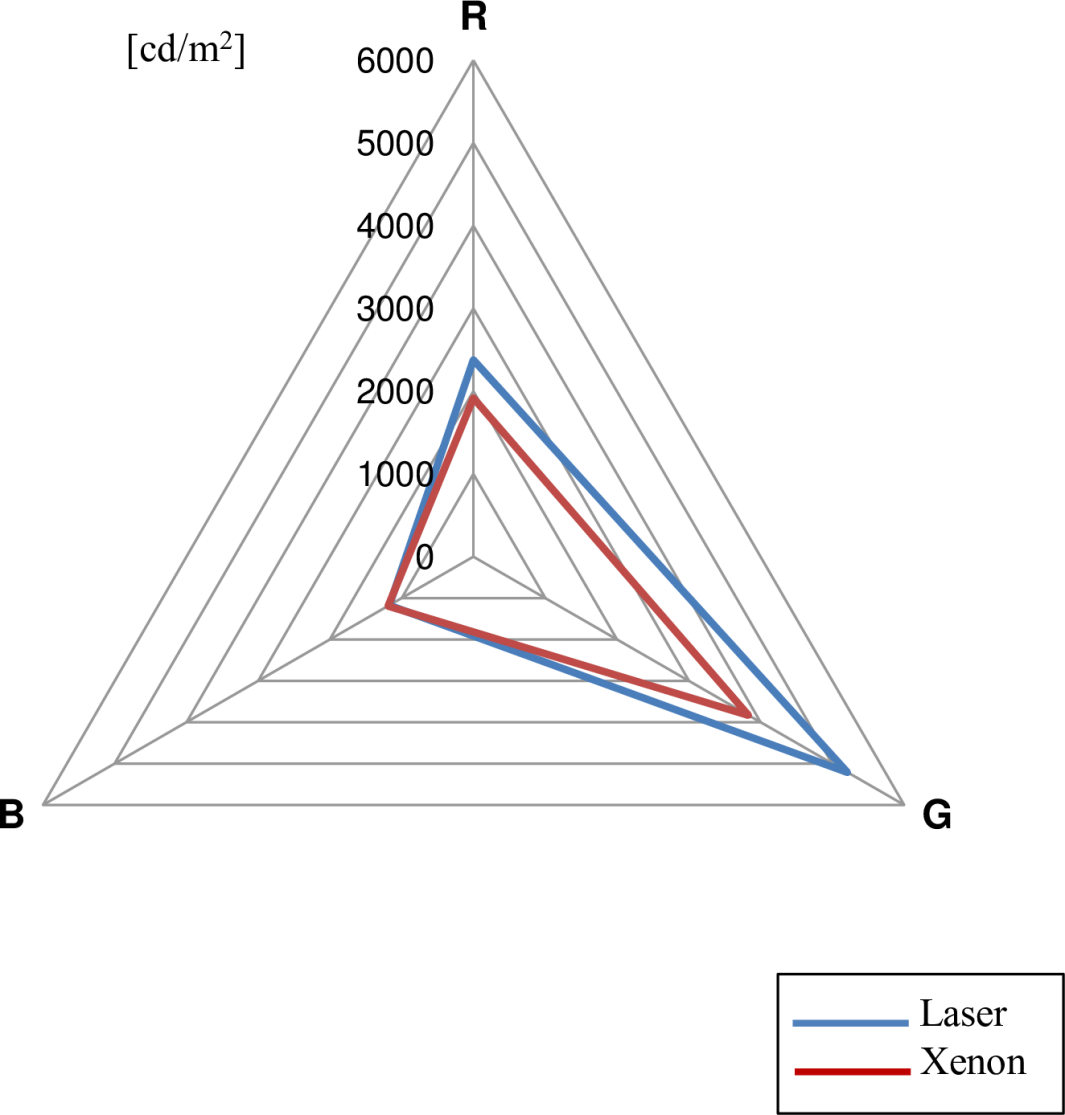
Radar chart showing the range of luminance of both xenon and laser lights. The laser light has a wider range of brightness than the xenon light, especially along the red and green color axes (cd/m^2^ = candela per square meter).

A luminance meter was used to measure the luminance and the chromaticity (x and y coordinates) for each color of a color sample chart illuminated by xenon or laser light (Table 1). The coordinates were plotted over the CIE1931 color space chromaticity diagram, and the differences between the two light sources were observed (Fig 3). The color range of the laser light was much wider, especially in the green and red color areas. The luminance values of the red, green, and blue colors were plotted on a radar chart, and it was found that the laser light expressed a wide range of luminance along the red and green axes. The luminance range over the blue axis was almost identical (Fig 4).

**Table 1:** Examples of luminance meter readings.

	Xenon			Laser		
Color	Luminance (cd/m^2^)	x coordinate	y coordinate	Luminance (cd/m^2^)	x coordinate	y coordinate
**Red**	1920	0.521	0.357	2380	0.529	0.334
**Green**	3830	0.311	0.482	5210	0.211	0.598
**Blue**	1190	0.198	0.185	1170	0.174	0.192

The three main colors from the color sample chart (red, green, and blue) are shown. Readings include the luminance in candela per square meter (cd/m^2^) and chromaticity (x and y coordinates) for xenon and laser light sources.

### Visual fatigue status assessment

The results of the visual fatigue assessment are summarized in Tables 2 and 3 (S2 File). Accommodative function and lacrimal secretion tests did not show significant differences in visual fatigue status between before and after laser microscope use. The ocular symptoms VAS score analysis showed that xenon caused more itchiness and frequent blinking than laser light. For the remaining 13 symptoms, there were no significant differences from xenon usage.

**Table 2:** Visual fatigue assessment results: HFC-1 and strip meniscometry.

	Xenon		Laser		
	Before	After	Before	After	p-Value
**Strip meniscometry (mm)**	3.4 ± 1.7	2.9 ± 1.3	3.7 ± 1.9	2.6 ± 1.2	0.862
**Changes of HFC-1 value**	53.9 ± 8.4	53.8 ± 8.6	53.2 ± 3.8	52.7 ± 3.7	NS

HFC-1, high-frequency component in the low accommodation group; NS, not significant.

**Table 3:** Visual fatigue assessment results: VAS scores.

	Xenon		Laser		
	Before	After	Before	After	p-value
**Feeling of heat**	0.96 ± 1.99	1.79 ± 2.05	1.40 ± 2.53	1.61 ± 2.44	NS
**Itchiness**	1.35 ± 2.65	1.69 ± 2.51	1.62 ± 3.01	1.46 ± 2.71	<0.01*
**Foreign body sensation**	1.90 ± 2.91	2.17 ± 3.11	1.87 ± 3.13	1.79 ± 2.95	NS
**Tearing**	0.71 ± 1.01	0.73 ± 0.87	0.90 ± 1.64	1.09 ± 1.69	NS
**Frequent blinking**	2.19 ± 2.72	2.78 ± 3.16	3.12 ± 2.51	2.26 ± 2.51	0.0438*
**Congestion**	1.60 ± 2.36	2.12 ± 2.82	2.67 ± 3.20	2.61 ± 3.15	0.0584
**Eye pain**	1.74 ± 2.52	1.90 ± 3.21	2.38 ± 3.12	2.42 ± 3.55	NS
**Droopy eyelid**	1.98 ± 2.56	2.25 ± 3.06	2.61 ± 3.27	2.79 ± 3.45	NS
**Dry eye**	2.28 ± 2.83	3.44 ± 3.05	3.61 ± 2.83	2.88 ± 3.42	0.221
**Blurred vision**	1.74 ± 2.59	2.17 ± 2.72	1.97 ± 2.30	3.08 ± 3.09	0.124
**Diplopia**	0.89 ± 1.67	1.06 ± 1.23	1.75 ± 2.25	2.48 ± 2.63	0.833
**Difficulty in sustaining visual activity**	0.60 ± 1.03	1.43 ± 1.64	2.24 ± 2.45	2.99 ± 3.00	NS
**Seeing flashes**	0.50 ± 0.86	1.23 ± 1.90	1.23 ± 2.10	1.41 ± 1.86	NS
**Obscured vision**	0.74 ± 1.30	1.05 ± 1.07	2.28 ± 2.42	1.93 ± 2.74	0.0616
**Headache**	1.64 ± 2.51	1.90 ± 2.86	1.69 ± 2.73	1.66 ± 2.80	NS

NS, not significant.

### Measurement of temperature change

**Fig 5:**
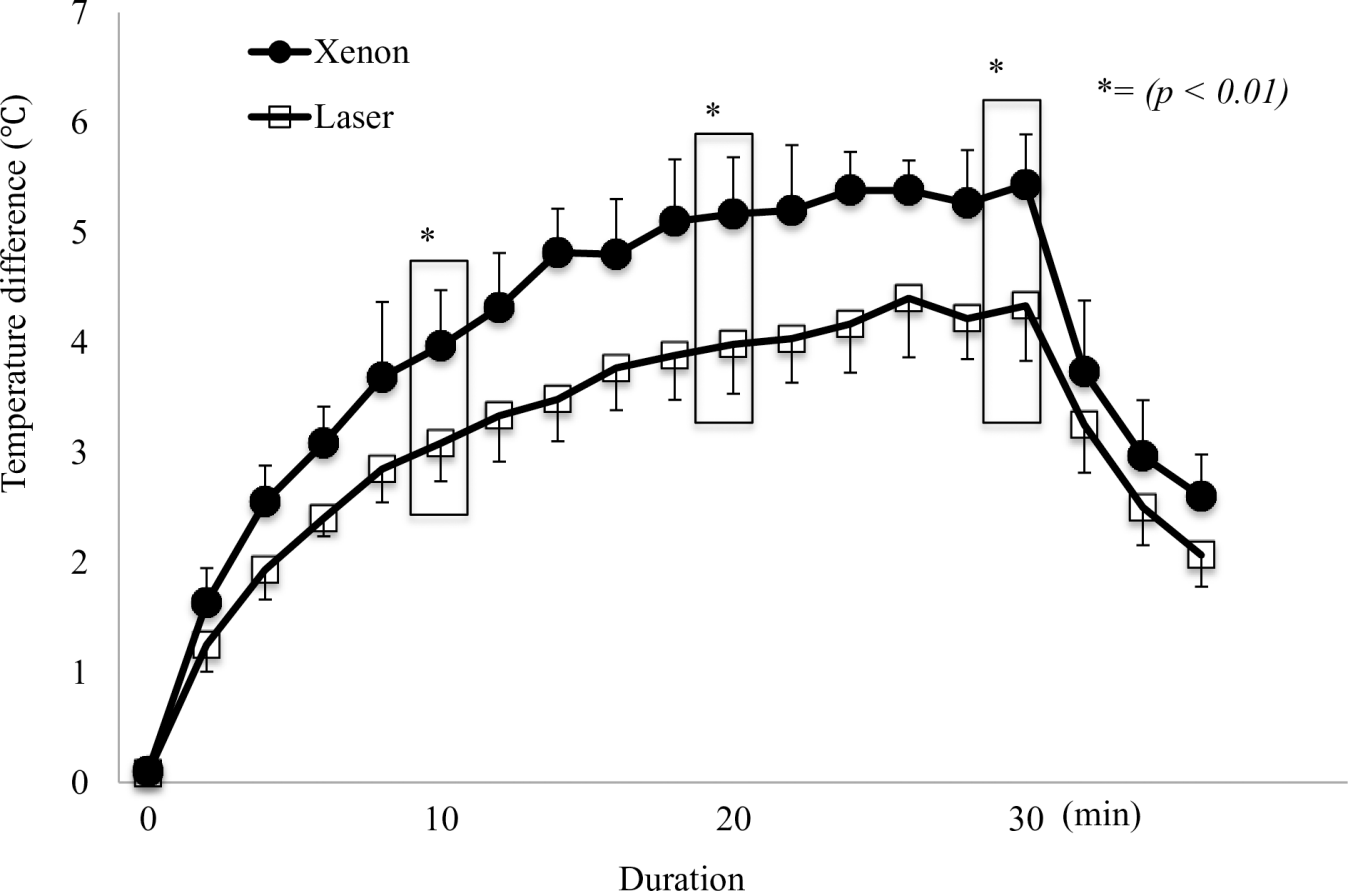
Illumination of each muscle sample by laser or xenon light at 150,000 lx for 30 min. The temperature of the muscle under the xenon light was significantly higher than that under the laser light after 10, 20, and 30 min (all p < 0.01).

The temperature of the muscle under the xenon light was significantly higher than that under the laser light after 10, 20, and 30 min (all p < 0.01) (Fig 5) (S2 File).

## Discussion

Over the past 20 years, xenon lamps have been the most commonly used light sources in neurosurgical microscopes. Bright illumination is a prerequisite for precise and efficient observation of the deep part of the brain parenchyma, especially at high magnification. While the xenon light offers a high-powered light source, it raises the focal temperature of the tissues and can result in focal thermal injury. The wavelength spectrum of a xenon lamp includes the strong energy intensity of the 365-nm ultraviolet light wavelength [1]. Irradiation with ultraviolet light increases reactive oxygen species in living cells, which can cause bleb development on cell surfaces, potentially resulting in neuronal cell death within 24 h [1]. Irradiation by a xenon light source within the limit allowed by safety regulations (200 mW/cm^2^) can result in an increased skin surface temperature of 43°C [2,24]. Hashimoto et al. reported that the use of saline over the cortical surface drastically decreased the severity of neuronal damage and the energy level of light emitted from surgical microscopes [1].

The laser light source was developed by Gould in 1959, and it has been used in many fields since a bright illumination with low energy consumption can be obtained (Table 4) [25]. Although the directivity and coherence of the laser light sources were sufficient to illuminate the operative field, the coherence posed a risk to the human body. To avoid such light coherence, a new laser light source diffuser panel that reduces the coherency to safe levels for surgeons’ eyes and patients’ neural tissues was developed. In the current study, the newly developed laser light showed a lower focal temperature than that of xenon light.

**Table 4:** Comparison between different light sources.

	Halogen	Xenon	LED	Laser
**Directivity**	Poor	Poor	Poor	Good
**Color rendering**	Good	Good	Average	Average
**Color calibration**	Poor	Poor	Average	Good
**Energy efficiency**	Poor	Poor	Good	Good
**Life span**	Poor	Poor	Good	Good

LED, Light Emitting Diode.

The laser light source might be expensive due to hardware and manufacturing costs are high. According to the manufacturer, the laser light source has a life expectancy 40 times longer than a xenon light source. Although the retail price of the laser light source is almost five times that of a xenon light source, the maintenance and replacement costs are much cheaper than that of a xenon light source. We assume in the long run the laser light source price can be equivalent to the xenon light source.

Chromaticity analysis revealed that the laser light has a broader range of color coordinates and a wider range of brightness than xenon light. This allows for greater color discrimination and saturation (vividness).

Three ophthalmological methods were used to assess visual fatigue (VAS, HFC-1 values, and strip meniscometry). Regarding the ciliary muscle accommodation microfluctuation test and the eye tear test, there were no significant differences between the eye status before and after using the laser light. Additionally, the ocular symptom VAS score revealed that xenon caused itchiness and frequent blinking more than laser light. For the remaining 13 symptoms, no differences between laser and xenon light were observed. The HFC-1 values remained within the normal range after using the laser microscope. The combined results of these three tests suggest that laser light is safe for the user’s eyes.

The focal temperature on the surface illuminated by laser light had a significantly lower temperature than that illuminated by xenon light. This is primarily attributed to the lack of the high-energy wavelengths of ultraviolet rays and probably to the ability to illuminate the surgical field with a lower level of illuminance than required for xenon light. In this study, it was subjectively shown that laser light could achieve the same degree of xenon light luminance with a lower illumination, e.g., 120,000 lx.

Matsuda et al. reported that the estimated time for 50% photobleaching was doubled for laser light compared to xenon light [10]. They found that a laser light source was able to inhibit the photobleaching during fluorescence image-guided surgery for malignant gliomas. They had used in his study the same light source we developed. Indocyanine green is a fluorescence substance that is excited under near-infrared light in the blood vessels and some types of tumors. The laser light source does include the 785 nm light’s wavelength needed for indocyanine green excitation. Therefore, it may be useful to measure the fluorescence properties in cerebrovascular surgery and brain tumor surgery in the future.

There are some limitations to the current study. First, the number of available volunteers was insufficient for a reliable comparison between the two different technologies. Second, because the measurement of temperature changes was not performed in vivo, it remains unclear if the results can be translated into significant differences in actual clinical practice. Third, considering it is the first model, our laser light source system is probably not perfect. The laser generator size is large and not installed within the microscope. The manufacturer is working on a newer model, which shall be smaller than the present one. Further clinical trials are necessary to validate the impact of this new light source on the patient’s outcome and prognosis.

## Conclusion

We developed an efficient and safe laser light source for surgical microscopes. The novel laser light source has several advantages over conventional sources, namely a lack of harmful ultraviolet waves, a longer lifespan, a lower focal temperature than that of other light sources, a wide range of brightness and color production, and improved safety for the user’s vision.

## Supporting information

S1 File Visual fatigue evaluation using a visual analogue scale. Scores are converted from the point marked on a 10-cm-long line, which represents the severity of subjective symptoms (minimum, 0 cm; maximum, 10 cm). Volunteers placed a mark corresponding to the severity of each symptom.
(TIF)

S2 File The experiment’s data. (PDF)
