# Supplementary figures and images for: Utility and safety of a novel surgical microscope laser light source
Source: PLoS One. 2018 Feb 1;13(2):e0192112. doi: 10.1371/journal.pone.0192112 (PMC5794154; doi:10.1371/journal.pone.0192112)

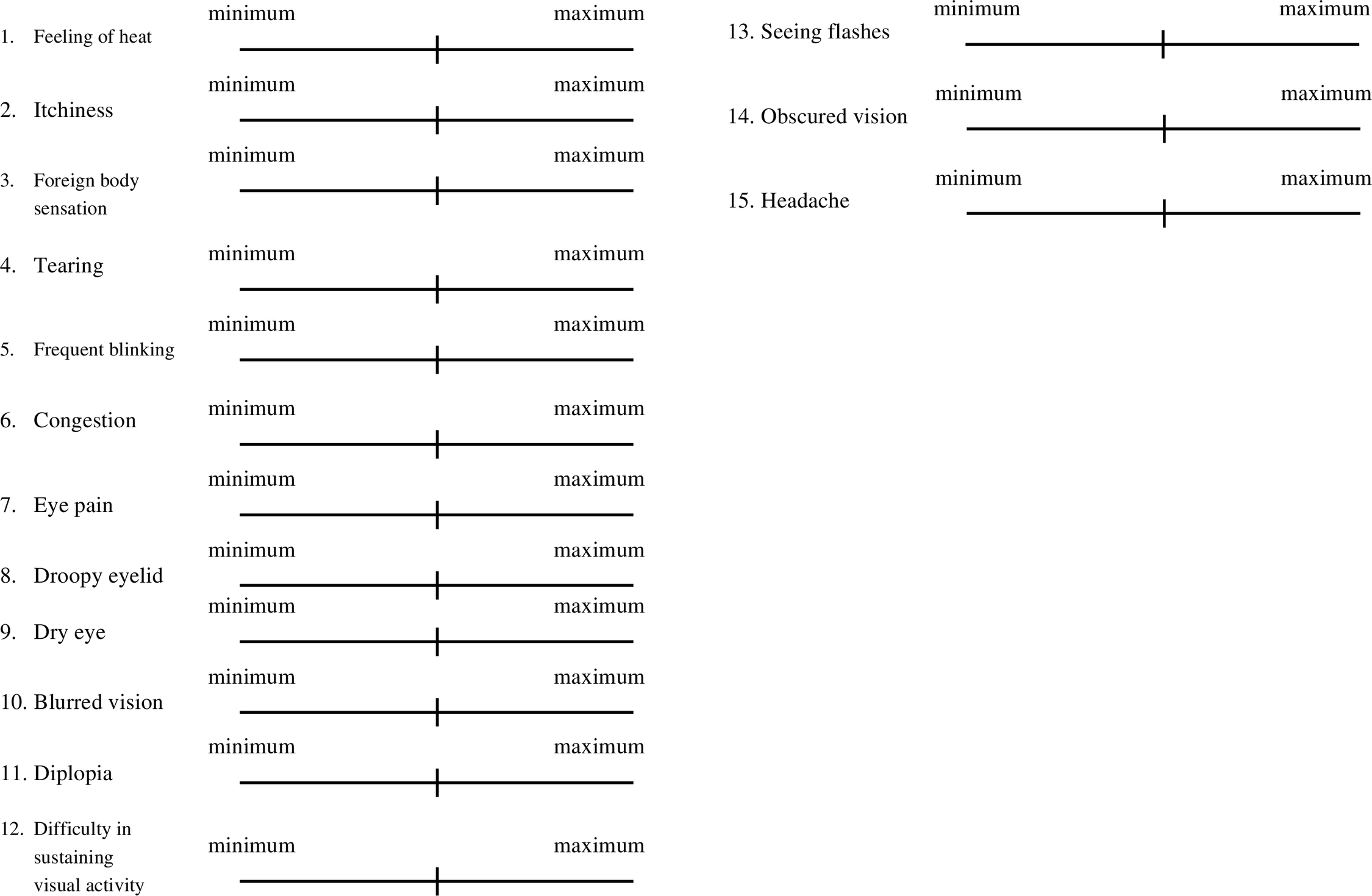

Supplement: S1 File — Scores are converted from the point marked on a 10-cm-long line, which represents the severity of subjective symptoms (minimum, 0 cm; maximum, 10 cm). Volunteers placed a mark corresponding to the severity of each symptom. (TIF) [file pone.0192112.s001.tif]
