# Supplementary material for: Utility and safety of a novel surgical microscope laser light source
Source: PLoS One. 2018 Feb 1;13(2):e0192112. doi: 10.1371/journal.pone.0192112 (PMC5794154; doi:10.1371/journal.pone.0192112)
Supplement: S2 File — (PDF) [file pone.0192112.s002.pdf]

Luminance visual comparison test

volunteer No.	Age, Sex	Luminance (lx)
1	31, F	148,000
2	33, M	180,000
3	22, M	120,000
4	30, F	120,000
5	23, M	180,000
6	28, F	120,000
7	31, M	120,000
8	29, F	148,000
9	27, M	180,000
10	33, F	220,000
11	36, F	180,000
12	30, M	120,000
13	33, M	120,000
14	27, M	180,000

Visual fatigue status assessment

			Laser light				Xenon light			
volunteer			strip meniscometry		HFC-1 value		strip meniscometry		HFC-1 value	
No.	Age, Sex	side	before	after	before	after	before	after	before	after
15	29, M	R	3	2	54.78	54.42	1	1	52.34	54.24
		L	5	4	58.11	55.51	2	3	52.03	52.98
16	30, M	R	3	1	60.05	52.56	2	2	50.99	54.12
		L	2	1	54.34	58.33	2	1	49.37	47.81
17	36, F	R	5	3	55.46	52.60	2	4	59.54	56.06
		L	9	2	52.99	57.30	2	2	50.42	46.37
18	21, M	R	4	2	49.36	51.52	5	5	49.55	51.67
		L	5	1	47.37	47.42	4	3	51.31	52.83
19	31, M	R	4	5	53.97	50.60	6	5	53.44	50.85
		L	5	1	51.53	50.60	5	3	53.12	51.45
20	32, M	R	1	4	57.33	54.91	4	3	87.53	87.70
		L	1	3	53.20	53.50	3	1	52.20	52.72
21	39, M	R	5	5	59.93	59.35	2	3	58.60	60.18
		L	4	3	55.87	53.39	8	4	57.89	58.72
22	21, M	R	3	2	50.07	56.64	3	2	53.82	52.74
		L	5	2	52.39	55.14	3	2	53.06	52.48
23	21, M	R	3	3	50.22	47.10	5	3	46.57	50.12
		L	5	3	46.39	47.13	3	5	50.09	44.41
24	35, M	R	1	2	51.63	49.14	4	4	48.75	49.97
		L	1	3	49.79	47.52	2	2	47.16	47.59

Visual fatigue assessment (Laser light)

volunteer	Test 1		Test 2		Test 3		Test 4		Test 5		Test 6		Test 7		Test 8		Test 9		Test 10		Test 11		Test 12		Test 13		Test 14		Test 15	
No.	before	after	before	after	before	after	before	after	before	after	before	after	before	after	before	after	before	after	before	after	before	after	before	after	before	after	before	after	before	after
15	7	6	7	6	7	7	7	6	36	6	10	5	8	5	9	6	9	5	9	4	9	8	7	10	7	50	7	13	7	10
16	4	20	3	3	4	3	3	50	22	50	50	50	22	3	72	73	2	2	2	50	65	50	64	75	20	25	65	20	23	3
17	56	40	70	59	76	67	51	34	21	38	70	62	79	74	72	72	73	68	16	61	23	44	20	58	27	25	29	21	64	56
18	0	0	0	0	0	0	0	0	27	18	0	0	0	0	15	21	50	23	0	0	0	0	0	0	0	0	0	0	0	12
19	1	0	0	0	0	18	0	0	0	18	1	0	0	0	0	0	0	22	24	0	0	0	0	0	0	0	0	1	0	0
20	0	0	0	0	0	0	0	0	0	0	0	0	0	0	10	0	50	0	0	0	0	0	0	0	0	0	0	0	0	0
21	0	0	0	0	0	0	0	0	50	0	0	0	1	0	0	0	24	0	24	0	0	0	25	0	0	0	23	0	0	0
22	72	79	82	77	85	84	29	19	88	81	86	72	78	88	82	92	85	95	72	70	28	75	58	34	69	41	54	88	75	85
23	0	16	0	1	15	0	0	0	18	15	0	0	0	0	1	15	18	0	0	49	0	21	0	72	0	0	0	0	0	0
24	0	0	0	0	0	0	0	0	50	0	50	72	50	72	0	0	50	73	50	74	50	50	50	50	0	0	50	50	0	0

Visual fatigue assessment (Xenon light)

	Test 1		Test 2		Test 3		Test 4		Test 5		Test 6		Test 7		Test 8		Test 9		Test 10		Test 11		Test 12		Test 13		Test 14		Test 15	
volunteer	before	after	before	after	before	after	before	after	before	after	before	after	before	after	before	after	before	after	before	after	before	after	before	after	before	after	before	after	before	after
No.	before	after	before	after	before	after	before	after	before	after	before	after	before	after	before	after	before	after	before	after	before	after	before	after	before	after	before	after	before	after
15	0	3	0	3	0	3	0	3	0	3	0	2	2	2	0	3	0	20	0	19	0	3	0	3	0	16	0	3	0	4
16	4	18	4	12	4	11	4	14	4	14	4	19	5	25	25	23	5	20	5	13	50	32	25	34	8	22	25	25	50	49
17	28	24	54	54	55	62	24	24	43	58	55	64	60	68	57	69	61	64	52	25	32	23	27	21	27	23	39	20	58	60
18	0	0	0	0	0	0	0	0	23	22	0	0	0	0	20	21	20	17	0	0	0	0	0	0	0	0	0	0	0	0
19	0	1	0	0	0	0	0	0	0	19	0	1	0	2	0	1	0	12	0	13	0	1	0	0	0	0	0	10	0	0
20	0	49	0	1	0	2	0	1	0	1	0	1	0	0	0	1	0	0	0	1	0	1	0	16	1	0	0	0	0	1
21	0	0	0	0	0	0	22	0	0	0	0	0	0	0	20	0	0	0	0	0	0	0	0	0	0	0	0	0	0	0
22	64	59	77	74	81	89	21	19	84	93	51	74	57	93	76	92	80	94	67	80	7	25	8	19	14	62	10	26	56	76
23	0	25	0	25	0	0	0	12	15	68	0	1	0	0	0	15	12	67	0	66	0	21	0	50	0	0	0	21	0	0
24	0	0	0	0	50	50	0	0	50	0	50	50	50	0	0	0	50	50	50	0	0	0	0	0	0	0	0	0	0	0

Measurement of temperature change

Time (minute)	Laser 1	Laser 2	Laser 3	Laser 4	Laser 5	Laser 6	Xenon 1	Xenon 2	Xenon 3	Xenon 4	Xenon 5	Xenon 6
0	0.2	0.0	-0.1	0.3	0.1	0.0	0.0	0.0	0.0	0.2	0.2	0.2
2	1.6	1.4	1.4	1.0	0.9	1.2	1.3	1.9	1.4	1.8	1.3	2.1
4	2.0	2.1	2.4	1.8	1.6	1.7	2.2	2.7	2.3	2.5	2.4	3.2
6	2.5	2.7	2.2	2.3	2.4	2.3	2.6	2.9	3.1	2.9	3.4	3.6
8	2.9	2.7	3.3	2.9	2.3	3.0	2.6	3.8	3.5	3.4	3.9	4.9
10	3.2	2.8	3.5	3.1	2.5	3.4	3.5	4.1	3.8	3.5	3.9	5.0
12	3.6	3.0	4.0	3.3	2.7	3.4	4.3	4.0	4.0	4.2	4.0	5.4
14	3.5	3.5	4.1	3.4	2.8	3.6	4.7	5.1	4.7	4.2	4.7	5.5
16	3.7	3.6	4.3	3.8	3.1	4.1	5.3	4.7	4.7	4.3	4.2	5.6
18	3.9	3.7	4.5	4.2	3.2	3.8	4.8	4.7	5.4	4.5	5.0	6.2
20	4.0	3.8	4.7	3.6	3.4	4.4	5.3	5.0	5.4	4.6	4.6	6.1
22	3.9	3.7	4.6	4.0	3.5	4.5	5.1	5.1	5.5	4.8	4.4	6.3
24	4.2	3.8	4.9	4.4	3.5	4.2	5.2	5.5	5.7	4.9	5.1	5.9
26	4.7	4.5	5.0	4.6	3.3	4.3	5.2	5.5	5.6	5.0	5.2	5.8
28	4.1	4.0	4.9	4.3	3.7	4.3	5.4	5.4	5.2	4.5	5.0	6.1
30	4.5	4.1	5.0	4.1	3.5	4.8	5.5	5.1	5.4	5.1	5.1	6.4
32	3.2	2.7	3.7	3.8	2.7	3.4	3.6	3.2	4.4	3.4	3.0	4.8
34	2.5	2.0	3.1	2.7	2.3	2.4	2.7	3.1	2.4	2.9	2.7	4.0
36	2.2	1.5	2.3	2.3	1.9	2.2	2.3	2.4	2.2	2.5	2.9	3.3
